# Successful management of a large feline skin defect using xenogeneic bovine amniotic membrane: a case report

**DOI:** 10.1186/s12917-026-05389-9

**Published:** 2026-03-25

**Authors:** Alaa Waleed, Howaida Abu-Ahmed, Mostafa Kassem, Hoda Elkhenany

**Affiliations:** Department of Surgery, Faculty of Veterinary Medicine, Alexandria University, Qetaa an Nahdah, Moharam Bek, Alexandria, Governorate 5410012 Egypt

## Abstract

**Background:**

Amniotic membrane has gained interest as a biological dressing due to its antimicrobial, anti-inflammatory, and pro-regenerative properties. However, its short shelf life and limited availability often constrain its clinical use in veterinary medicine. This report describes, for the first time, the use of bovine-derived AM preserved in a 1% propolis medium as a xenogeneic biological dressing for the management of extensive skin necrosis in a cat.

**Case presentation:**

An eight-month-old female domestic cat (1.9 kg, unvaccinated) presented with severe dorsal skin sloughing and purulent discharge following subcutaneous fluid therapy for calicivirus infection. The wound involved approximately 12–15% of the body surface area. Initial management included systemic and topical antibiotics with daily cleansing for 5–7 days until the wound bed appeared dry and clean. Bovine AM preserved in propolis at −20 °C for 21 days was applied once the wound was ready for biological coverage. A second AM application was performed 30 days later using a membrane preserved for ~50 days. Quantitative analysis showed progressive wound closure from 97.1 cm² at baseline to complete epithelialization by Day 33 and full functional recovery with hair regrowth by Day 74. No recurrence, infection, or excessive scarring was observed.

**Conclusion:**

This case demonstrates that propolis-preserved bovine AM can serve as a practical, biologically active xenogeneic graft promoting rapid wound healing in cats. The findings suggest that propolis is a promising natural preservative that maintains AM bioactivity during storage.

## Background

Large full-thickness skin defects in small animals represent a significant therapeutic challenge due to limited available skin for closure, high risk of infection, and delayed healing. Conventional treatments such as skin grafting or secondary intention healing may be associated with prolonged recovery, scar formation, or donor site morbidity [1].

Amniotic membrane has gained increasing attention as a biological dressing owing to its anti-inflammatory, antimicrobial, and epithelialization-promoting properties. Its extracellular matrix composition provides a suitable microenvironment for cell migration, angiogenesis, and collagen deposition, making it an attractive option for managing complex wounds [2, 3].

Propolis, a natural resinous compound produced by honeybees, possesses potent antioxidant and antimicrobial activities [4–6], hence can serve as an effective preservative medium that maintains the biological integrity of amniotic membrane during storage.

The present case report describes the successful management of an extensive skin defect in a cat using propolis-preserved amniotic membrane applied twice over a one-month interval. This report highlights the clinical efficacy, practicality, and healing dynamics associated with this approach, supported by both quantitative wound area measurements and gross morphological evaluation.

## Case description

An eight-month-old female domestic cat (1.9 kg) was presented to the clinic in June 2025 with severe purulent discharge from the subcutaneous tissue and extensive skin sloughing over the dorsolateral trunk, affecting approximately 12–15% of the total body surface area. The lesion developed a few days after the cat had received a large single-dose subcutaneous (SC) fluid therapy administered during treatment for a Calicivirus infection. On clinical examination, the cat was lethargic, hyperthermic, and exhibited markedly reduced appetite. The affected skin appeared wet, fragile, and friable with extensive exudation, consistent with necrosis and secondary infection.

### Initial management prior to amniotic membrane application

The initial goal of treatment was to stabilize the cat, control infection, and prepare the wound bed for subsequent biological dressing. Systemic antibiotic therapy was initiated with amoxicillin–clavulanic acid (Hibiotic 228 mg/5 mL; 15 mg/kg, q12h) for five consecutive days. Local wound management included cleansing the area twice daily using sterile saline and povidone–iodine solution, followed by amikacin spray applied directly over the exposed subcutaneous layer without removing the affected skin.

**Fig. 1:**
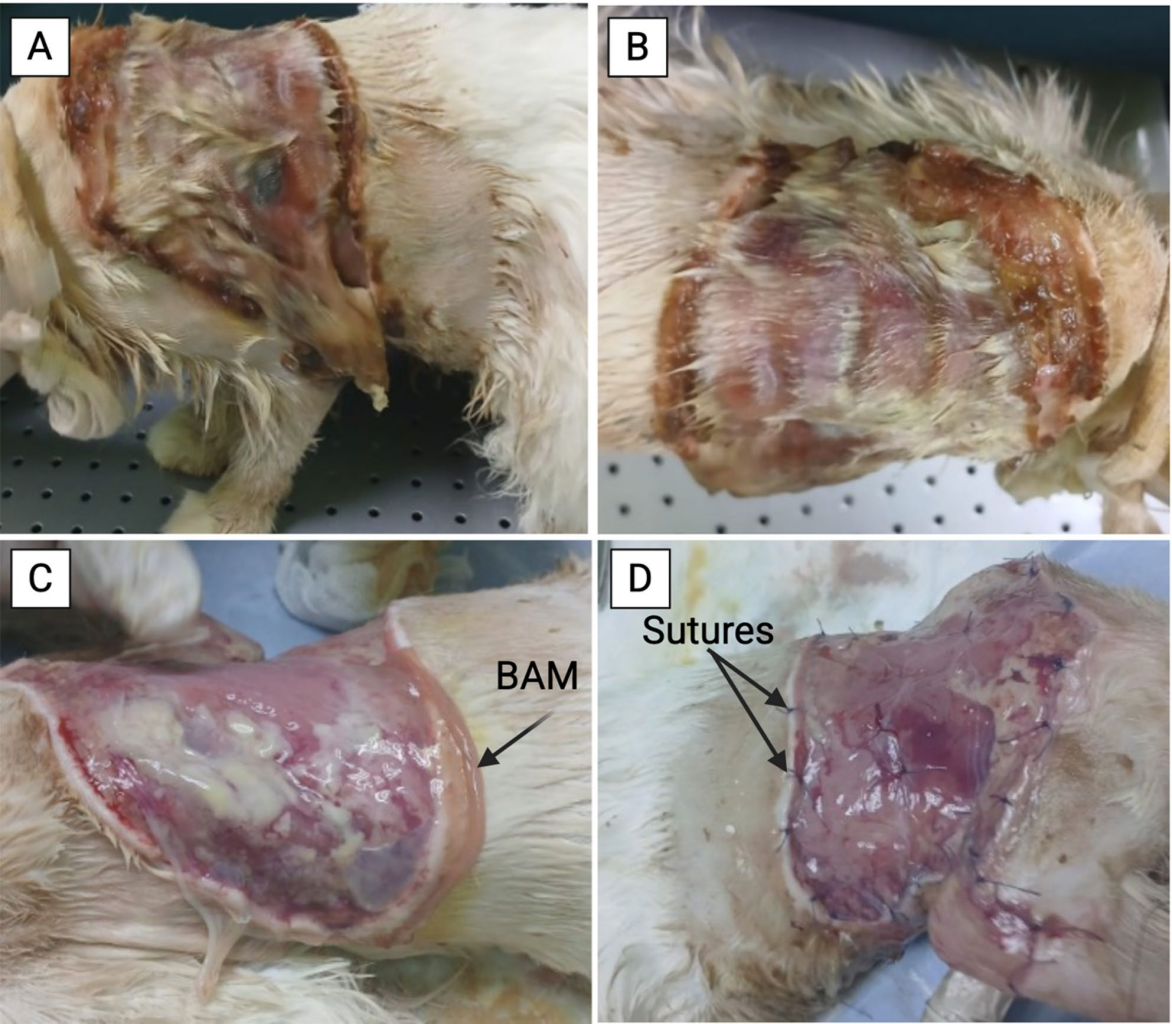
Clinical progression and amniotic membrane application. **A, B** The cat at the first day of treatment, showing extensive skin sloughing with wet, fleshy, and fragile tissue. **C, D** Application of the bovine amniotic membrane and its fixation using a simple interrupted suture pattern

This initial treatment phase lasted approximately 5–7 days. During this period, the wound evolved from a wet, fragile, and infected surface to a dry, thin, and dense one, indicating a reduction in infection and readiness for amniotic membrane application. The necrotic skin was not removed at the early stage to avoid dehydration, excessive fluid loss, or risk of secondary infection, especially given the cat’s debilitated condition and the owner’s initial hesitance toward surgical intervention (Fig. 1).

### Amniotic membrane preparation and preservation

The bovine amniotic membrane was obtained from a clinically healthy donor cow originating from a regularly monitored dairy herd under veterinary authority supervision. Herd health management included routine vaccination against brucellosis (RB51), infectious bovine rhinotracheitis (IBR marker live vaccine), and bovine viral diarrhea (BVD killed vaccine), along with periodic screening using the Rose Bengal field test for brucellosis. Membranes were collected under strict aseptic conditions immediately after parturition, thoroughly rinsed with sterile saline to remove blood clots and debris, and processed within 6 h of delivery to minimize microbial contamination.

The bovine amniotic membrane (BAM) was preserved using a glycerol-based medium supplemented with propolis (Shana Company, Egypt). Briefly, raw propolis powder (Shana Company, Egypt) was weighed and suspended at a concentration of 1% (w/v) in sterile phosphate-buffered saline (PBS). The suspension was stirred continuously for 12–24 h at room temperature, gently warmed to 37 °C to enhance solubilization, and then filtered through sterile gauze to remove insoluble debris. This 1% propolis suspension was incorporated at 30% (v/v) into a glycerol-based preservation solution, resulting in a final composition of 70% glycerol and approximately 0.3% (w/v) propolis. The membrane was stored at − 20 °C until use. Glycerol served as the primary cryoprotective and dehydrating agent, while propolis served as a natural antimicrobial and antioxidant additive. The membrane used in this case was prepared on 26 May 2025 and had been preserved for 21 days prior to application.

The preparation and clinical use of the preserved BAM were performed under an Institutional Animal Care and Use Committee approved protocol (IACUC, Alexandria University; Approval No. AU013202503081612).

### Amniotic membrane application and clinical outcome

The first BAM application was performed on June 15, 2025, after wound debridement and cleaning. The cat was fasted for 6–8 h before anesthesia using xylazine (0.5 mg/kg), ketamine (5 mg/kg), and propofol (30 IU every 30 min). Necrotic tissue was removed, wound edges were refreshed, and BAM preserved in 1% propolis was applied, two small pieces (6 × 7 cm) and one large piece (7 × 15 cm), with the chorion side facing the wound (Fig. 1). The membrane was sutured in place and covered with Vaseline-coated gauze and sterile wrapping. Postoperative care included ceftriaxone (50 mg/kg IV, 5 days), metronidazole (15 mg/kg IV), and meloxicam (0.2 mg/kg SC). Daily dressing changes were performed. MEBO^®^ cream (Moist Exposed Burn Ointment; Julphar, UAE), composed primarily of sesame oil, beeswax, and herbal extracts containing β-sitosterol (0.25%) and berberine, was applied topically once daily to maintain a moist wound environment and support epithelial regeneration. Amikacin spray (Acdima trading, Egypt) was applied topically once daily to prevent bacterial infection. An E-collar was used to prevent self-trauma.

Following the first application, rapid improvement in wound appearance was observed, with progressive reduction in exudation and healthy granulation tissue formation evident within the first week. A second BAM application was performed on day 30 to further promote epithelialization and enhance tissue remodeling, using a single small piece from the same batch. The wound appeared clean and sterile, with no need for local antibiotics. Oral amoxicillin–clavulanic acid (15 mg/kg, twice daily for 5 days) and meloxicam (0.2 mg/kg, single dose) were administered post-application. Healing accelerated markedly after the second treatment, with rapid epithelialization and complete closure achieved within days, accompanied by full hair regrowth and restored skin texture.

### Outcome and follow-up

Quantitative monitoring of wound healing was performed using standardized digital photographs of the wound taken at each time point with a ruler placed adjacent to the wound for scale calibration. Wound areas were measured using ImageJ software (NIH, USA), and the percentage of wound reduction at each time point was calculated relative to the initial wound area on Day 0 using the formula: *Wound reduction (%) = [(A₀ − Aₜ) / A₀] × 100*, where A₀ is the initial wound area and Aₜ is the wound area at time t. The daily healing rate (%/day) was calculated as the change in wound reduction between two time points divided by the number of days between measurements.

**Fig. 2:**
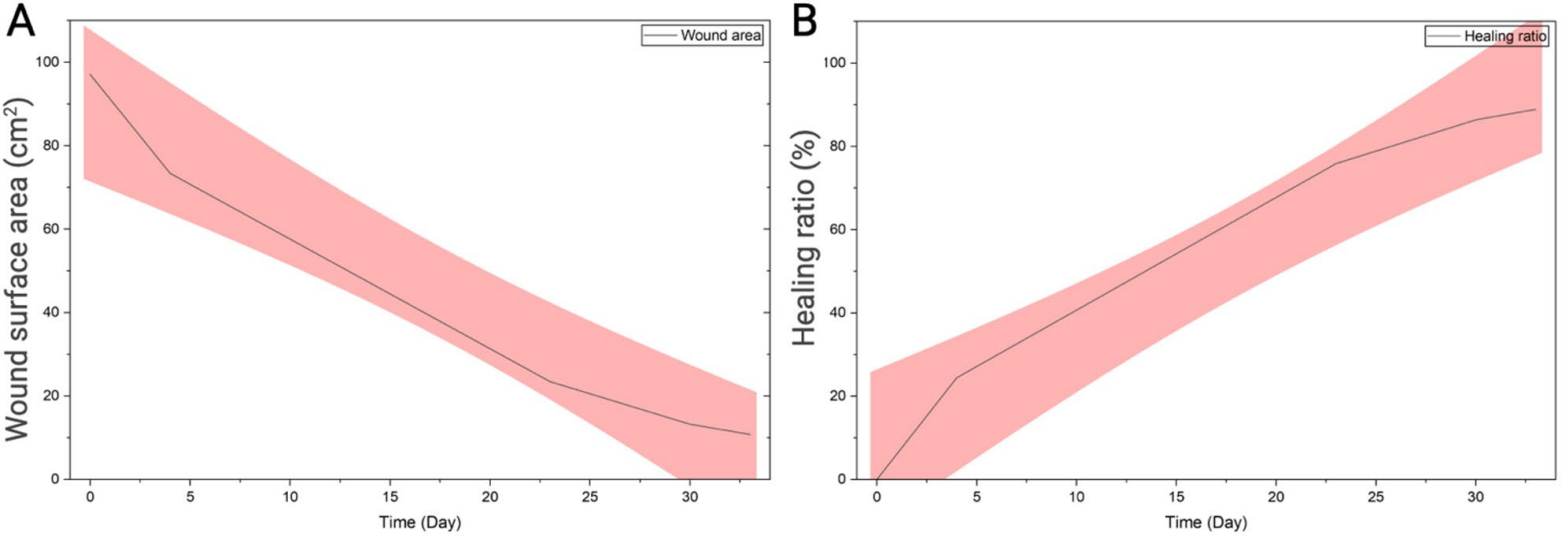
Quantitative assessment of wound healing over time. **A** Wound surface area (cm²) measured at sequential time points from Day 0 (time of first bovine amniotic membrane application) to Day 33 using ImageJ software. Images were captured with a ruler as a size reference. **B** Healing ratio (%) calculated at each time point and normalized to the initial wound area at Day 0

The initial wound area of 97.09 cm² decreased progressively, achieving 24.4% healing by Day 4, 75.9% by Day 23, and 86.4% by Day 30. The calculated daily healing rate was highest during the early phase (≈ 6.1% per day from Day 0–4), then progressively declined to ≈ 2.7% per day between Day 4–23 and ≈ 1.5% per day between Day 23–30 (Table 1). This reduction in healing velocity, together with the persistence of a residual defect, prompted a second amniotic membrane application at Day 30. Complete epithelialization and functional skin restoration were achieved by Day 33, with a total healing ratio of 88.9% (Fig. 2). The treated area demonstrated full closure with complete hair regrowth, absence of infection or necrosis, and no evidence of excessive scarring. The cat regained normal appetite, activity, and overall health, confirming systemic recovery and good tolerance of the treatment protocol.

**Table 1:** Quantitative assessment of wound healing progression

Day	Wound area in cm2	Healing ratio (%)	Daily healing rate (%)/day
Day 0	97.093	—	—
Day 4	73.375	24.43	6.11
Day 23	23.446	75.85	2.71
Day 30	13.229	86.37	1.5

**Fig. 3:**
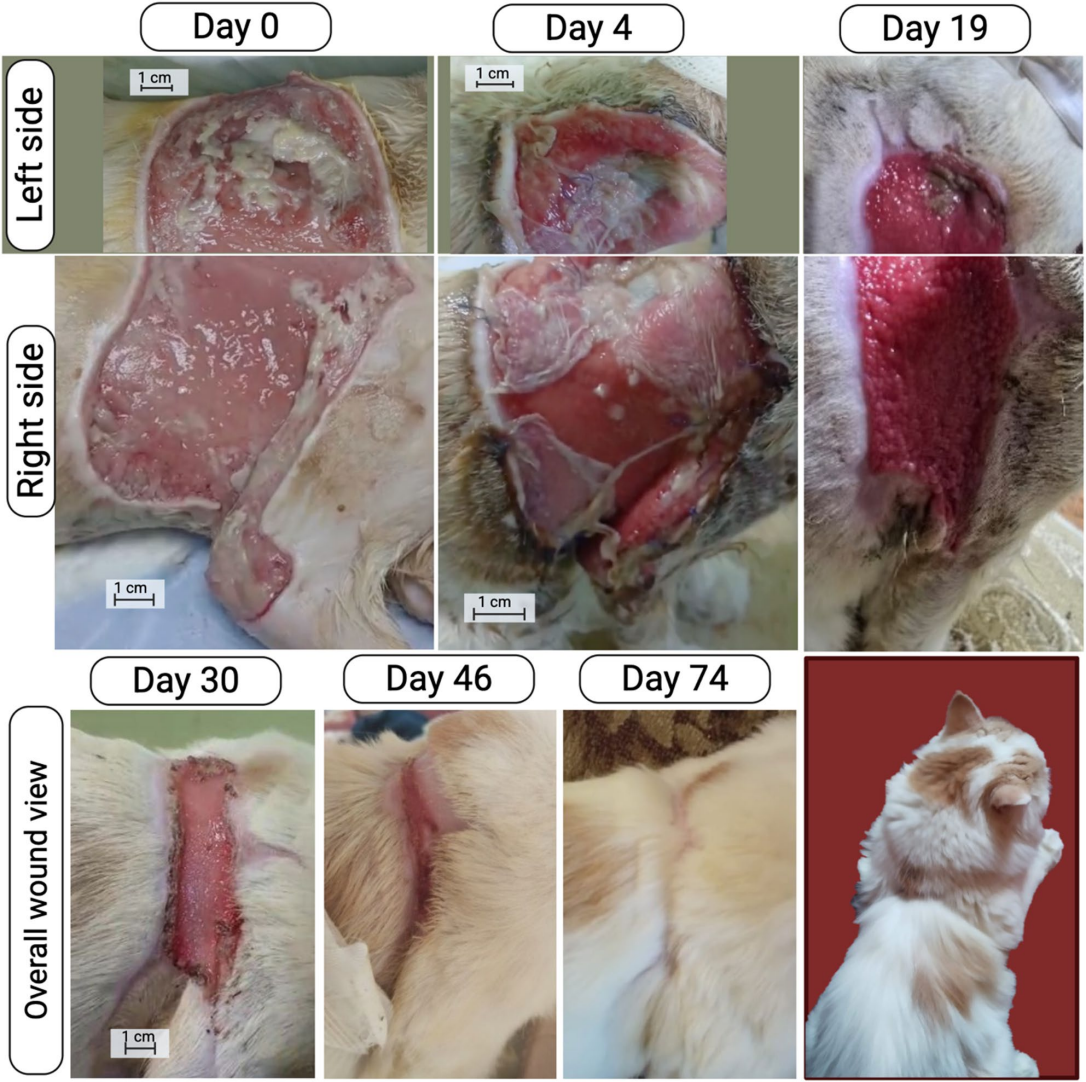
Wound healing progression over time. Representative photographic images showing wound healing progression over time. The upper panel presents the right and left wound sites separately at days 0, 4, and 19 post injury. The lower panel shows the full wound surface area from the left side, demonstrating progressive reduction in wound size at days 30, 46, and 74. Scale bar = 1 cm for days 0, 4 and 30)

Sequential macroscopic evaluation supported these findings, revealing an orderly progression of tissue repair (Fig. 3). On Day 0, the wound displayed extensive skin loss, exposed subcutaneous tissue, and purulent exudation, characteristic of acute inflammation and necrosis. By Day 4, exudation had diminished, and early granulation tissue with pink-red coloration indicated neovascularization and infection control. By Day 19, the wound surface was largely covered by healthy, well-vascularized granulation tissue with visible epithelial migration at the edges. After the second amniotic membrane application (Day 30), the tissue became smoother and paler, signaling transition to the remodeling phase. By Day 46, a thin epithelial layer bridged the wound, and by Day 74, the defect was completely closed with uniform epithelialization and hair regrowth, demonstrating full structural and functional restoration. Notably, the second application associated with a shift from granulation tissue to organized epithelial remodeling, suggesting that repeated membrane application may support sustained regeneration in large wounds where a single application may be insufficient.

## Discussion

In the present case, the use of propolis-preserved amniotic membrane applied twice (Day 0 and Day 30) was associated with rapid granulation tissue formation and near-complete epithelialization by Day 33, with full functional skin restoration and hair regrowth by Day 74. These findings are consistent with the known biological actions of amniotic membrane namely, promotion of keratinocyte migration, angiogenesis, modulation of inflammation, and provision of an extracellular matrix scaffold that facilitates cell ingrowth, which have been described in both reviews and experimental wound models [7].

In the present case, it is important to note that the amniotic membrane used was derived from a bovine source, i.e., a xenogeneic graft relative to the feline recipient. The choice of BAM offers logistical and practical advantages, specifically, larger available surface area, easier sourcing, and lower cost compared to autologous or homologous (feline or human) membranes. Indeed, in recent experimental models, BAM has been shown to promote full-thickness skin wound healing in dogs. For example, a study reported that fresh BAM significantly accelerated re-epithelialization, angiogenesis, collagen remodelling and hair follicle regeneration compared to control wounds in dogs [8]. Another mouse model found that BAM produced significant reductions in wound area, enhanced expression of wound-healing markers (SMAD2/3, α-SMA), and improved collagen-III deposition compared to standard dressings [9].

Although propolis has not previously been investigated as an additive to amniotic membrane preservation media, its biological properties make it a promising candidate. Studies have shown that propolis can serve as a natural fixative preserving tissue morphology, maintain cell viability and gene expression in preserved cells, and act as an effective natural preservative due to its antimicrobial and antioxidant capacity. These findings support the hypothesis that propolis could maintain the biological integrity and sterility of amniotic membranes during storage [10–12]. Herein, the preserved membrane was thoroughly rinsed with sterile saline prior to application to minimize any direct topical effect of propolis. Therefore, the observed wound healing is unlikely to be due solely to residual propolis; however, the propolis may have conditioned the membrane matrix during preservation, contributing to sustained bioactivity after implantation. This aligns with our conceptual goal of using the amniotic membrane as a scaffold for the delivery of bioactive compounds.

Repeat amniotic membrane application is an accepted clinical strategy in some settings (notably ophthalmology) and is recommended by commercial amniotic membrane wound-care protocols when continued coverage or reinforcement of healing is required; applying a second amniotic membrane at Day 30 may therefore have supported the transition from the proliferative to the remodeling phase and enhanced epithelial maturation in this large defect [13, 14].

However, direct comparative data in veterinary large-defect skin wounds are limited. Case reports and small series in small animals report variable healing durations after amniotic membrane therapy (often several weeks to months depending on wound chronicity, size, and adjunctive care), and few published reports specifically evaluate the effect of repeated amniotic membrane applications on healing kinetics [15]. When compared to published studies using xenogeneic BAM, our case demonstrates both quantitative and qualitative outcomes at least equal to, and in some respects superior to, those described. For example, in a canine full-thickness skin wound study using BAM, complete closure was achieved by 5 weeks in the lyophilized group [16].

In our case, near-complete epithelialization (≈ 88.9% reduction) was recorded by Day 33 and final closure by Day 74, despite a large (~ 12–15% body surface) and infected wound. Additionally, the combination of BAM preservation in propolis supplemented medium, and a second application after 30 days, may have contributed to the enhanced healing kinetics. Given that the literature does not appear to include planned multiple applications of AM in large veterinary skin defects, this case adds new clinical evidence to the field and supports the feasibility of xenogeneic BAM use in feline cutaneous wound repair.

Therefore, although the favorable time course in the current case (≈ 33 days to epithelialization) suggests that the use of amniotic membrane preserved in propolis supplemented medium and its repeated application contributed meaningfully to the outcome, this single-case observation cannot prove superiority over standard care. The absence of tissue rejection is consistent with the known low immunogenicity and immunomodulatory properties of the amniotic membrane, but direct measurement of immune response was not performed. Moreover, the individual contributions of the membrane versus propolis cannot be fully separated in a single-case design. Controlled studies or a case series comparing single versus multiple amniotic membrane applications, ideally with standardized wound sizing and objective endpoints, would be needed to confirm the degree to which double application improves healing speed or quality and to evaluate the relative contribution of incorporated bioactive compounds.

Despite the xenogeneic origin of the bovine amniotic membrane, no clinical evidence of tissue rejection was observed throughout the healing period. The absence of clinical or macroscopic signs of tissue rejection in the present case is consistent with the known immunological characteristics of amniotic membrane. Amniotic membrane exhibits very low immunogenicity due to minimal expression of major histocompatibility complex (MHC) class I antigens and negligible expression of MHC class II antigens, as well as the absence of vascular structures, which together limit immune recognition and inflammatory cell infiltration [17]. In addition, amniotic membrane has been shown to exert immunomodulatory and anti-inflammatory effects through the release of bioactive mediators that suppress pro-inflammatory cytokine production and regulate macrophage and lymphocyte activity [18]. These properties have enabled the successful clinical use of amniotic membrane across species barriers, including xenogeneic applications, without eliciting overt rejection responses in both experimental models and clinical settings [16, 19]. The lack of rejection observed in this feline case following bovine amniotic membrane application is therefore in agreement with previously reported biological behavior of amniotic membrane based grafts.

### Limitations

This report represents a single clinical case, and direct experimental confirmation of amniotic membrane bioactivity following storage in a propolis supplemented glycerol medium was not performed. The favorable outcome likely reflects the combined effects of the amniotic membrane scaffold and propolis supplementation, a clinically relevant strategy given the limited applicability of propolis alone to small wounds.

## Conclusion

The repeated application of BAM preserved in propolis supplemented glycerol medium successfully promoted rapid and complete healing of a large skin defect in a cat. This approach effectively controlled infection, supported granulation, and enhanced epithelialization, resulting in full functional and cosmetic recovery. The outcome suggests that xenogeneic amniotic membrane can serve as a safe and practical biological dressing for managing extensive wounds in veterinary practice. 

## Data Availability

All data generated or analyzed during this study are included in this published article.
